# ABC Transporters B1, C1 and G2 Differentially Regulate Neuroregeneration in Mice

**DOI:** 10.1371/journal.pone.0035613

**Published:** 2012-04-24

**Authors:** Toni Schumacher, Markus Krohn, Jacqueline Hofrichter, Cathleen Lange, Jan Stenzel, Johannes Steffen, Tina Dunkelmann, Kristin Paarmann, Christina Fröhlich, Annekathrin Uecker, Anne-Sophie Plath, Alexandra Sommer, Thomas Brüning, Hans-Jochen Heinze, Jens Pahnke

**Affiliations:** 1 Neurodegeneration Research Laboratory (NRL), Department of Neurology, Universities of Rostock and Magdeburg, Magdeburg, Germany; 2 German Center for Neurodegenerative Diseases (DZNE), Magdeburg, Germany; 3 Leibniz Institute for Neurobiology (LIN), Magdeburg, Germany; Sanford-Burnham Medical Research Institute, United States of America

## Abstract

**Background:**

ATP-binding cassette (ABC) transporters are essential regulators of organismic homeostasis, and are particularly important in protecting the body from potentially harmful exogenous substances. Recently, an increasing number of *in vitro* observations have indicated a functional role of ABC transporters in the differentiation and maintenance of stem cells. Therefore, we sought to determine brain-related phenotypic changes in animals lacking the expression of distinct ABC transporters (ABCB1, ABCG2 or ABCC1).

**Methodology and Principal Findings:**

Analyzing adult neurogenesis in ABC transporter-deficient animals *in vivo* and neuronal stem/progenitor cells *in vitro* resulted in complex findings. *In vivo*, the differentiation of neuronal progenitors was hindered in ABC transporter-deficient mice (ABCB1^0/0^) as evidenced by lowered numbers of doublecortin^+^ (−36%) and calretinin^+^ (−37%) cells. *In vitro*, we confirmed that this finding is not connected to the functional loss of single neural stem/progenitor cells (NSPCs). Furthermore, assessment of activity, exploratory behavior, and anxiety levels revealed behavioral alterations in ABCB1^0/0^ and ABCC1^0/0^ mice, whereas ABCG2^0/0^ mice were mostly unaffected.

**Conclusion and Significance:**

Our data show that single ABC transporter-deficiency does not necessarily impair neuronal progenitor homeostasis on the single NSPC level, as suggested by previous studies. However, loss of distinct ABC transporters impacts global brain homeostasis with far ranging consequences, leading to impaired neurogenic functions *in vivo* and even to distinct behavioral phenotypes. In addition to the known role of ABC transporters in proteopathies such as Parkinson's disease and Alzheimer's disease, our data highlight the importance of understanding the general function of ABC transporters for the brain's homeostasis and the regeneration potential.

## Introduction

ABC transporters arise from one of the largest known superfamily of genes, and can be found in every living organism. Most ABC transporters carry out unidirectional transmembrane transport [1]. Functionally, ABC transporters can be separated into three groups: i) *importers*, mostly used to import nutritious molecules and ions in prokaryotes [2], [3], ii) *exporters*, which secrete a broad variety of dietary cytotoxic agents and thereby also numerous therapeutic drugs across biological membranes (reviewed in [4]), and iii) a subgroup that is involved in chromatin reorganization, DNA repair, telomere maintenance and RNA trafficking [5], [6], [7]. Currently, 49 different human ABC genes have been identified in humans, and these are classified into seven subgroups named ABCA to ABCG based on amino acid sequence-similarities and phylogeny (reviewed in [8], [9]). ABC transporters are capable of transporting a wide range of peptides, hydrophobic molecules, drugs or drug conjugates. Together, they form a chemo-defense network against xenobiotics [10]. Recently, ABC transporters have been discovered to exhibit important export functions for proteins involved in Alzheimer's disease [11], [12], with ABCC1 being of exceptional importance for the clearance of β-amyloid from the brain [13]. In addition to their role as crucial components of barriers that protect the brain from toxic substances, such as the blood-brain barrier (BBB) and the blood-cerebrospinal fluid barrier (reviewed in [12], [14]), these molecules also are expressed on stem cells of various tissues, and were discovered in cancer cells.

Accordingly, p-glycoprotein, later identified as ABCB1, was first found to maintain cell surface permeability in a hamster cell line [15], and was later linked to chemoresistance in a multidrug resistant Chinese hamster ovary cell line [16]. Later, ABCG2 was isolated from multidrug-resistant breast cancer cells in the absence of ABCB1 [17], while ABCC1 was found to be highly expressed in a group of hematological and solid tumors (reviewed by [18]). Due to the fact that many cancer cells manifest a stem cell- or progenitor-like gene expression pattern [19], it was not surprising that ABC transporter expression has been detected in a variety of organ-specific stem cells throughout the organism (reviewed in [20]). Hematopoietic stem cells show a reduced staining profile for the mitochondrial marker Rhodamine 123, which is a substrate of ABCB1, indicating high expression levels of ABCB1 in these stem cells [21]. Using fluorescence-activated cell sorters and bone marrow cells stained with fluorescent dyes such as Rhodamine 123 and Hoechst 33342, a small, low-profile subset of cells with phenotypic markers of hematopoietic stem cells was revealed [22]. This population is lost in the Hoechst profile after administration of the ABCB1 inhibitor verapamil [22], [23], and was later confirmed in non-hematopoietic cell populations such as pancreatic mesenchymal stem cells [24]. Mice lacking ABCB1 or ABCG2 do not show altered phenotypes [25], [26] except for higher brain concentrations of ABCB1-transported agents/ligands, an increased immunological response to normal bacterial flora (ABCB1^0/0^) [27], and a distinct form of dietary-induced protoporphyria (ABCG2^0/0^) [28]; however, these studies did not investigate neurogenic or cognitive functions.

Recently, *in vitro* studies showed high expression rates of ABCB1 [29] and ABCG2 [30] in neurospheres during proliferation. The expression of both ABC transporters decreased substantially with the advancing state of differentiation, again suggesting a functional involvement of ABC transporters in stem cell homeostasis [31].

Considering the increasing number of observations indicating a functional role of ABC transporters in stem cell maintenance and differentiation, mostly from *in vitro* studies, we hypothesized that neurogenic processes would be impaired due to the deficiency of distinct ABC transporters. We focused on the transporters ABCB1 (MDR1, subfamily B) and ABCG2 (BCRP, subfamily G) based on the studies of Islam and colleagues [29], [30], who first observed the transient expression of ABC transporters in NSPCs. Furthermore, we chose ABCC1- (MRP1, subfamily C) deficient mice because little information is available about its function in neurogenic processes, and because we recently showed its potential significance for brain homeostasis in proteopathies such as Alzheimer's disease [17]. In the present study, we focused on the assessment of possible impairments of neurogenesis and cognitive alterations *in vivo* and cell-intrinsic effects on NSPC functions *in vitro* with regard to deficiency for the distinct ABC transporters.

After comparative analyses of ABC transporter-deficient mouse strains and tissue *in vivo* and *in vitro*, we can confirm that a deficiency of ABC transporters leads to distinct impairments in NSPC maturation and adult neurogenesis *in vivo*. However, the *in vitro* experiments to determine proliferation and differentiation changes of NSPCs revealed that the underlying processes are not related to single stem cells. Consistent with the neurogenic alterations, we also show that ABC transporter-deficiency leads to far-ranging functional changes, even resulting in distinct behavioral phenotypes in a set of basic behavioral tests.

## Results

### ABC transporter-deficiency affects neuronal differentiation of NSPCs

**Figure 1:**
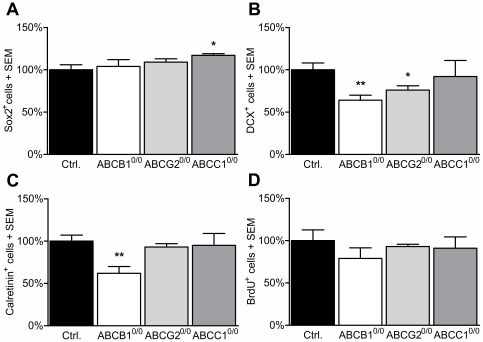
Deficiency of ABCB1 impairs neuronal differentiation in the SGZ of the dentate gyrus. Shown are the relative quantities of cells positive for Sox2, DCX, calretinin and BrdU in the SGZ of the dentate gyrus in ABC transporter-deficient mice as compared to the FVB controls. A) Sox2^+^ NSPCs increased significantly in ABCC1 transporter-deficient mice (+17%), while ABCB1- or ABCG2-deficient mice were unaffected. B) Early DCX^+^ neurons were significantly decreased in the absence of ABCB1 (−36%) and ABCG2 (−23%), while later, post-mitotic calretinin^+^ cells (C) were only reduced in ABCB1 transporter-deficient mice by 37%. D) The proliferation capacity (BrdU^+^ cells) was not affected in any of the analyzed ABC transporter-deficient mice. Error bar: SEM; * p<0.05; **p<0.01.

To assess the influence of ABC transporter-deficiency on neurogenesis, we analyzed molecular markers of neurogenic progenitors or immature neurons in the subgranular zone (SGZ) of the dentate gyrus (DG) in adult mice aged 3–4 months. We characterized marker proteins to quantify the differentiation states of NSPCs. To label very early stem and progenitor cells, we used an antibody against Sox2, a transcription factor that is required for maintenance of stem cell status, and therefore a reliable marker of early undifferentiated NSPCs [32], [33], [34]. To quantify the number of early neuronal progenitors, we used the marker protein doublecortin (DCX) [35], [36]. Postmitotic, newly generated neurons at a stage of axonal and dendritic targeting were labeled with an anti-calretinin antibody [37]. The quantification of the cells revealed a significant increase in Sox2^+^ early NSPCs only in ABCC1^0/0^ (+17%) mice, whereas their number was not altered in ABCB1^0/0^ or in ABCG2^0/0^ mice as compared to the genetic background controls (FVB/N) (Figure 1A). Investigating early neuronal progenitors, which are positive for DCX, revealed a significant decrease of these cells in mice lacking ABCB1 (−36%) or ABCG2 (−23%), but no changes in ABCC1^0/0^ mice (Figure 1B). Quantifying the number of post-mitotic, newly generated calretinin^+^ neurons, we also detected decreased cell numbers in ABCB1^0/0^ animals (−37%), while deficiency in ABCG2 or ABCC1 did not induce significant effects (Figure 1C). The proliferation capacity (BrdU^+^ cells) was not affected in any of the analyzed ABC transporter-deficient mice (Figure 1D). Thus, ABCC1^0/0^ mice only revealed slightly increased numbers of early NSPCs, while the fate of differentiation was not affected.

### ABC transporter-deficiency alters the rate of NSPC differentiation

**Figure 2:**
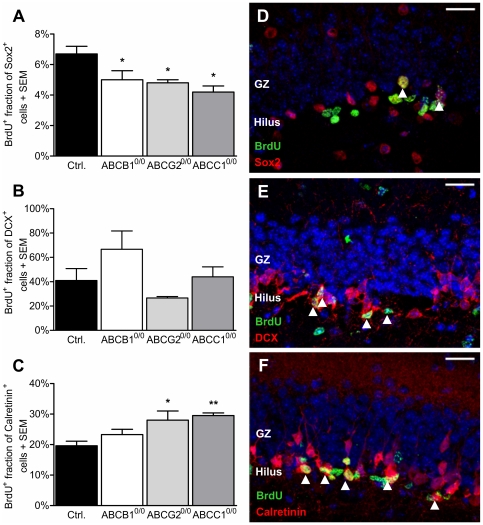
ABCC1 deficiency impairs stem cell proliferation and accelerates late neuronal differentiation. The graphs show the newly generated population (BrdU incorporation) of Sox2^+^ (A), DCX^+^ (B) and calretinin^+^ (C) cells in the SGZ of the DG. The number of proliferating Sox2^+^ NSPCs was significantly decreased in ABCB1- (−25%), ABCG2- (−28%) and ABCC1- (−37%) deficient mice, implicating slower proliferation of NSPCs within the 7 days of BrdU treatment. In contrast, late neuronal differentiation seems to be accelerated due to the significantly greater proportion of BrdU^+^ postmitotic early granule cells expressing calretinin in animals lacking ABCG2 (+43%) and ABCC1 (+51%) expression. The photomicrographs display expression examples for marker combinations: Sox2/BrdU (D), DCX/BrdU (E) and calretinin/BrdU (F). Arrowheads indicate double-labeled cells. Error bar: SEM; * p<0.05; **p<0.01; Scale bar: 20 µm.

To analyze Sox2, DCX and calretinin expression in the actively proliferating NSPC population, we double-labeled brain sections for BrdU and the respective marker protein. The mice received BrdU for 7 days prior to sacrifice. The analyses revealed decreasing amounts of newly proliferating Sox2^+^ NSPCs in the absence of each of the three ABC transporters (−25% ABCB1^0/0^; −28% ABCG2^0/0^; −37% ABCC1^0/0^; Figure 2A, 2D). The number of DCX^+^ neuronal progenitors did not show any ABC transporter-related changes in the proliferating population (Figure 2B, 2E). Interestingly, the population of post-mitotic, newly differentiated granule cells expressing calretinin significantly increased both in ABCG2^0/0^ (+43%) and ABCC1^0/0^ (+51%) mice, while the increase in ABCB1^0/0^ mice did not reach statistical significance (Figure 2C, 2F).

### ABC transporter deficiency does not impair neurogenic functions *in vitro*

**Figure 3:**
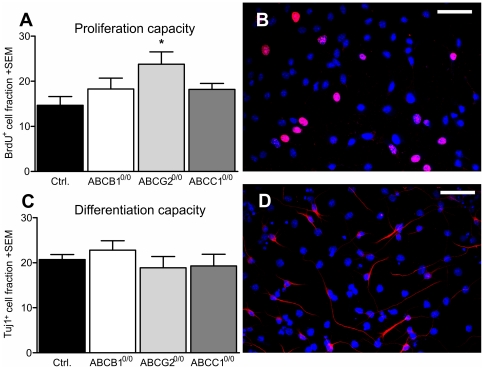
ABC transporter-deficiency did not lead to impairments of neuronal differentiation *in vitro*. ABCG2^0/0^ cells showed a significant increase in proliferation capacity (+62%, A) quantified by detection of BrdU uptake using immunofluorescence (B). The differentiation capacity was not impaired by loss of ABC expression (C) as revealed by quantification of neuronal cells positive for Tuj1 (D). Error bar: SEM; Scale bar: 50 µm.

To analyze the proliferation capacity of ABC transporter-deficient NSPCs, we used primary NSPC lines from the subventricular zone of control and ABC transporter-deficient animals. The proliferation capacity of NSPCs was measured by BrdU-uptake into the DNA of proliferating cells, and comparatively quantifying the BrdU^+^ cell fraction of each cell line. The BrdU intake over a period of 30 min revealed a significant increase in proliferation activity in cell lines deficient in the ABC transporter ABCG2 (+62%) (Figure 3A, 3B). The increase of proliferation activity in ABCB1^0/0^ and ABCC1^0/0^ cells did not reach significance.

To assess the differentiation potential of these primary NSPC lines, we let the cells differentiate for 3 days and quantified the number of differentiated neurons using immunofluorescence techniques. Comparing the fraction of Tuj1^+^, newly differentiated neurons among the different NSPC lines, we could not detect significant alterations (Figure 3C, 3D). Therefore we conclude that deficiency of ABCB1, ABCG2 or ABCC1 does not affect the ability of NSPCs to differentiate into neurons *per se*.

### ABC transporter expression in NSPCs

**Figure 4:**
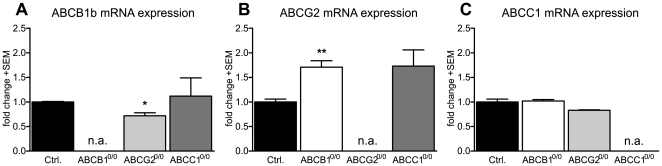
Expression of ABCB1b and ABCG2 are co-regulated in NSPCs *in vitro*. Relative mRNA expression of ABCB1b, ABCG2 and ABCC1 was measured against mRNA expression of GAPDH using TaqMan® assays. Expression of ABCB1b was significantly impaired (−28%) in ABCG2^0/0^ cells (A). In reverse, expression of ABCG2 was increased significantly (+71%) in animals lacking ABCB1 expression, while a comparable effect on ABCC1^0/0^ cells did not reach statistical significance (B). For ABCC1 mRNA expression, no significant changes were observed in the other cell lines (C). Error bar: SEM; * p<0.05; **p<0.01.

To confirm whether ABC transporters are expressed in proliferating NSPCs, we used TaqMan® assays to quantify relative expression levels for ABCB1a, ABCB1b, ABCG2 and ABCC1 in background controls and ABC transporter knockout strains. Quantitative RT-PCR analyses with NSPC mRNA confirmed that all three transporters (B1, C1, G2) are expressed, and also indicated a possible co-regulation between ABCB1 and ABCG2. We found only one form (ABCB1b) of the murine ABCB1 transporter expressed in NSPCs, while ABCB1a mRNA was not detectable. Analyses showed that the lack of ABCG2 led also to a significant decrease of ABCB1 mRNA amounts (−28%) (Figure 4A). In contrast, loss of ABCB1b expression led to a marked increase in ABCG2 mRNA (+71%) (Figure 4B). We also found ABCG2 mRNA levels increased by 73% as a result of ABCC1 deficiency, although this increase did not reach statistical significance (Figure 4B). On the other hand, ABCC1 deficiency did not lead to significant effects on the expression of ABCB1 or ABCG2. However, ABCC1 expression itself seemed not to be altered by the loss of neither ABCB1 nor ABCG2 expression (Figure 4C).

### ABC transporter-deficiency reduces neurogenesis in disease states

**Figure 5:**
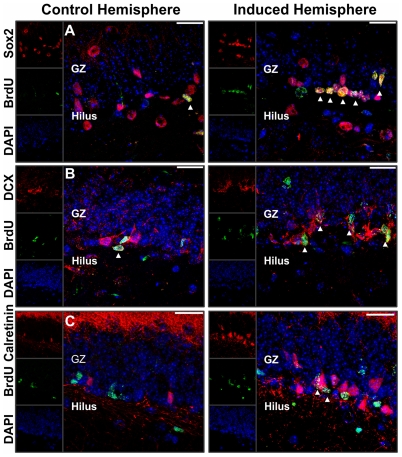
Neurogenesis is induced by controlled cortical impact (CCI) trauma. The photomicrographs show the SGZ of the DG after CCI (LEFT: Control hemispheres; RIGHT: hemispheres after CCI). Shown are the marker combinations Sox2/BrdU (A), DCX/BrdU (B) and calretinin/BrdU (C). Arrowheads indicate double labeled cells. Scale bar: 20 µm.

Traumatic brain injury is known to activate the proliferation and differentiation of resident NSPCs [38]. Since ABC transporter-deficiency under normal physiologic conditions causes only minor phenotypic changes, we wanted to assess the potential importance of the transporters during disease states. The controlled cortical impact (CCI) paradigm [39] was chosen to amplify minor effects of ABC transporter involvement in NSPC homeostasis with regard to proliferation and differentiation capacity. Therefore, cortical trauma was induced 7 days prior to analysis. In agreement with recent publications [38], [39], [40], neurogenic processes were significantly increased in the dentate gyrus of the induced (injured) hemisphere compared to the contralateral control hemisphere in all analyzed strains (Figure 5).

**Figure 6:**
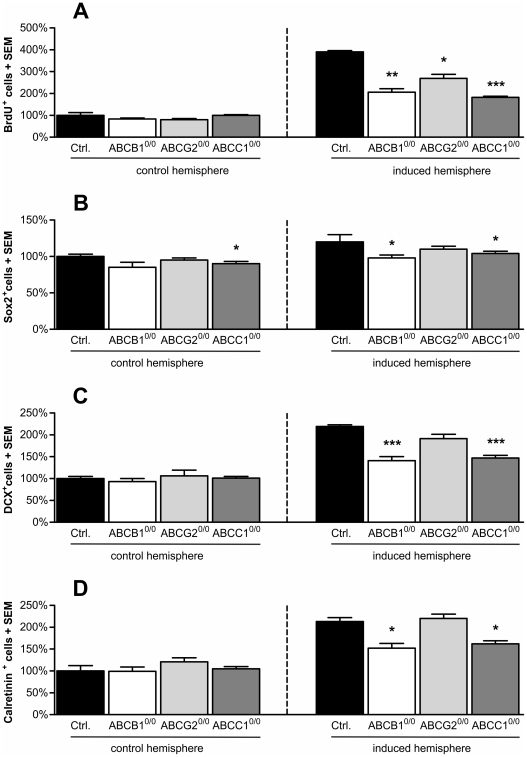
ABC transporters are important for neurogenic functions during disease states as revealed by the CCI paradigm. The proliferation capacity measured by BrdU incorporation (A) was significantly decreased in the induced hemisphere of all ABC transporter-deficient strains (ABCB1^0/0^: −47%; ABCG2^0/0^: −31%; ABCC1^0/0^: −53%) (right). The number of Sox2^+^ cells was not significantly altered in the induced brain hemisphere (B), but shows a trend toward a decreased number in ABCB1- and ABCC1- deficient mice that resembles the decreases in immature DCX^+^ and calretinin^+^ neurons (C, D). The number of DCX^+^ neuronal progenitors (B) was significantly reduced in the induced hemisphere of ABCB1- (−36%) or ABCC1-deficient (−33%) animals as compared to controls (right). Additionally, the number of early granule cells expressing calretinin (D) was decreased in the induced hemisphere of ABCB1- (29%) and ABCC1-deficient mice (−24%) (right), while there was no change in ABCG2-deficient mice. Error bars: SEM, *p<0.05; **p<0.01; ***p<0.001.

CCI-induced trauma resulted in a highly increased cell proliferation in the SGZ of the affected hemisphere in all analyzed mouse strains. The proliferation index of the ipsilateral hemispheres was significantly increased as compared to the contralateral, untreated control hemispheres in all mice. Importantly, ABC transporter deficiency substantially altered the extent of the proliferation increase. Relative to the control group, we observed significantly lower ipsilateral proliferation indices in all three ABC transporter-deficient strains (−47% ABCB1^0/0^; −31% ABCG2^0/0^; −53% ABCC1^0/0^, Figure 6A). Consistent with the highly increased cell proliferation, we also detected an expansion in the analyzed subpopulations after CCI-treatment. CCI led to increased numbers of Sox2^+^ NSPCs in the ipsilateral hemisphere independent of ABC transporter expression (Figure 6B). However, the CCI paradigm mostly affects the number of differentiating cells of the neuronal lineage that are DCX^+^ and calretinin^+^. All mice showed significantly more differentiating DCX^+^ neuronal progenitors in the induced hemisphere as compared to the control hemispheres. Comparing controls and ABC transporter-deficient strains, only ABCB1^0/0^ and ABCC1^0/0^ mice exhibited a substantial decrease in the number of differentiating DCX^+^ neuronal progenitors (−36% ABCB1^0/0^; −33% ABCC1^0/0^), while the cell number was not affected in ABCG2^0/0^ mice (Figure 6C). Analogous to the situation of the DCX^+^ cell pool, we also detected significantly lower numbers of calretinin^+^ postmitotic neurons in the induced hemisphere of ABCB1^0/0^ and ABCC1^0/0^ mice (−29% ABCB1^0/0^; −24 ABCC1^0/0^) (Figure 6D).

Comparing the different strains, we saw only a slight, non-significant decrease of Sox2^+^ progenitors in ABC transporter-deficient mice as compared to controls, indicating that the lack of ABCB1 or ABCC1 significantly reduces the number of cells differentiating into a neuronal lineage. The numbers of both DCX^+^ and calretinin^+^ cells were significantly lower in ABCB1- and ABCC1-deficient mice, while there was no difference observable in animals lacking the transporter ABCG2. The only parameter that was consistently affected in all three ABC transporter-deficient strains was the proliferation capacity.

### ABC transporter-deficiency does not affect neuronal area in the neocortex

**Figure 7:**
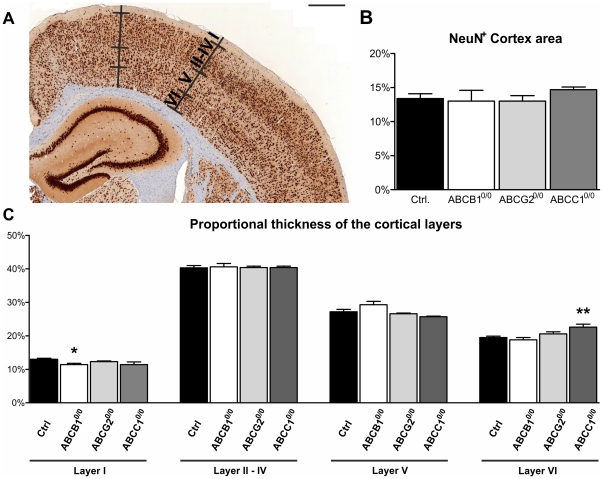
ABC transporter-deficiency influences cortical architecture of layers I and VI while neuronal density is not altered. Paraffin-embedded brain sections (A) were labeled with an antibody against NeuN, digitized, and the NeuN+ area was computer-assisted determined relative to the cortical area to quantify the neuronal density of the cortex (B). The thickness of the cortical layers I, II–IV (combined), V and VI were measured (A) and analyzed (C). The loss of ABCB1 expression significantly decreased the thickness of layer I (−12%) while loss of ABCC1 only affected the thickness of layer VI (+15%) Scale bars: 500 µm (A); Error bars: SEM, *p<0.05; **p<0.01.

The above analyses indicate that ABC transporter-deficiency differentially influences neurogenic function in the hippocampal subgranular zone. Thus, we wanted to determine whether these differences also influence the density of neurons in the neocortex. To assess neuronal area in the cerebral cortex, we established a reviewer-independent method to determine the total area covered by NeuN^+^ cells in a normalized cortical area. Paraffin-embedded brain sections were cut and immunohistochemically stained for the neuronal marker NeuN. Brain sections were digitized at a resolution of 230 nm/pixel, and the NeuN^+^ areas were discriminated from unstained cortex [41] to determine the NeuN^+^ fraction of the cortical area (Figure 7B). Comparison of the neuronal densities did not reveal significant differences between the ABC transporter-deficient strains and genetic background controls.

### ABC transporter-deficiency effects cortical cytoarchitecture

To assess whether loss of ABC transporter function possibly results in deficits in cortical development, which might be reflected in altered cortical architecture, we determined the thickness of the cortical layers I-VI. The NeuN-stained paraffin slices were measured according to Manuel and colleagues [42] for layer thickness (Figure 7A). By comparing the proportional size of layer I, II–IV (combined), V and VI, we found significant effects of deficient ABC transporter expression on cortical architecture in ABCB1^0/0^ and ABCC1^0/0^ strains. The loss of ABCB1 resulted in a decreased size (−12%) of layer I (Figure 7C), while all other layers were unaffected. The other significant effect showed ABCC1^0/0^ mice where only the size of layer VI was increased by 15% (Figure 7C). ABCG2^0/0^ animals showed no significant alterations in the cortical architecture. Layers I and VI represent the least cellular layers of the cortex with mostly interconnecting dendrites and axons.

### ABC transporter-deficiency influences activity and anxiety levels

**Figure 8:**
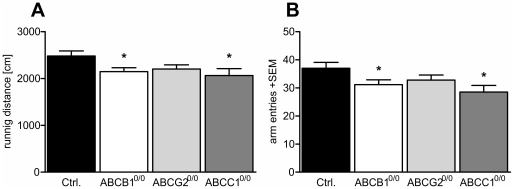
Lack of ABCB1 or ABCC1 expression leads to impaired exploratory activity. The diagrams show Y-maze-related parameters. Plotting the animals' course in the Y-maze, the running distance reveals a significant decrease in ABCB1- (−13%) and ABCC1- (−17%) deficient animals (A), while this parameter was only slightly, non-significantly decreased in ABCG2-deficient mice. The same behavioral pattern was detected for exploratory activity as assessed by the arm entries (ABCB1^0/0^ −16%; ABCC1^0/0^ −23%) (B). Error bar: SEM, *p<0.05.

Because the hippocampus functions as a main interface between different cortical inputs and is important for memory consolidation, we wanted to determine whether the absence of specific ABC transporters results in behavioral changes. To assess potential alterations in overall activity, exploratory behavior, or the level of anxiety, we used Y- and elevated plus mazes as well as the light/dark box setup [43], [44], [45], [46]. First, the animals were analyzed for their overall activity and changes in spontaneous alternation behavior using the Y-maze. We did not detect significant effects of transporter deficiency on alternation behavior, but the activity of ABCB1^0/0^ or ABCC1^0/0^ mice was significantly reduced as detected by the shortened running distance (Figure 8A) and reduced number of arm entries (Figure 8B). Specifically, ABCB1 deficiency led to a 13% decrease in the running distance and a 16% decrease in arm entries, and these effects were even more pronounced in ABCC1-deficient mice (running activity −17%, arm entries −23%).

**Figure 9:**
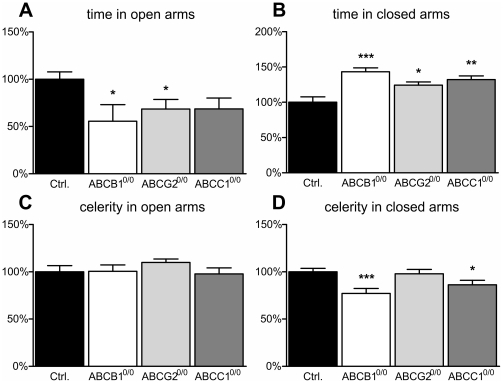
ABC transporter deficiency promotes anxiety. The diagrams show parameters assessed via the elevated plus maze. Mice deficient for ABCB1 (−44%) and ABCG2 (−32%) showed a significantly decreased presence in the open arms (A). The time of residence in the closed compartments was significantly increased in all ABC transporter-deficient mouse strains (ABCB1^0/0^ +43%; ABCG2^0/0^ +24%; ABCC1^0/0^ +32%) (B). Running speed (celerity) in the open arms was not changed in any of the ABC transporter-deficient mice (C). ABCB1 (−23%) and ABCC1 (−14%) transporter-deficient mice showed significantly lower celerity values in the closed arms (D). Error bar: SEM, *p<0.05; **p<0.01; ***p<0.001.

The elevated plus maze was used to assess anxiety levels in the mice. The basic principle of this maze is that the exploratory drive is equal for each arm, while the anxiety-driven aversion to enclosed arms is lower than to open arms, resulting in an increased exploration of the enclosed arms [47]. Analyzing the time spent in the open arms revealed a significant decrease in ABCB1^0/0^ or ABCG2^0/0^ mice (−44% ABCB1^0/0^; −31% ABCG2^0/0^) (Figure 9A), while the decline did not reach statistical significance in ABCC1^0/0^ mice. All transporter deficient mice remained longer in the enclosed compartments of the maze (+43% ABCB1^0/0^, +24% ABCG2^0/0^, +32% ABCC1^0/0^) (Figure 9B). Regarding the running velocity in the open compartment, we could not find any significant alterations (Figure 9C). In contrast, a significant reduction in running speed within the enclosed compartments was measured in ABCB1^0/0^ and ABCC1^0/0^ mice (−23% ABCB1^0/0^; −14% ABCC1^0/0^), but not in ABCG2^0/0^ mice (Figure 9D).

**Figure 10:**
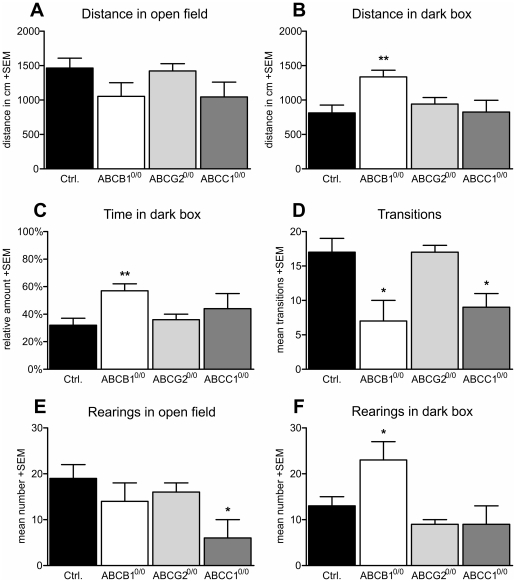
Light/dark box experiments verified altered anxiety and exploratory behavior. Comparing the running distance of the animals in the open field revealed no significant differences (A). However, the distance traveled by ABCB1-deficient mice (+64%) in the dark compartment (B) was significantly increased compared to the controls. Also, the time in the dark compartment (C) was significantly increased in ABCB1^0/0^ mice (+78%) compared to controls. The mean number of transitions between the two compartments (D) was impaired in both ABCB1^0/0^ (−59%) and ABCC1^0/0^ (−49%) mice. Comparing the rearing frequency in both compartments revealed a significant decrease for ABCC1^0/0^ (−67%) mice in the open field (E), while the frequency was increased in the dark compartment (F) for ABCB1^0/0^ (+71%) mice. Error bar: SEM, *<p0.05; **p<0.01.

The light/dark box experiments allowed us to assess the overall exploratory behavior as in an open-field test (running distance, rearing, transitions) as well as anxiety like behavior (light avoidance, differing activity under light and dark). Comparing the distances travelled by mice within the open compartment of the maze, no significant alterations were observed with regard to ABC transporter-deficiency (Figure 10A). However, mice deficient for ABCB1 showed increased activity within the dark compartment of the maze (Figure 10B), as indicated by increased running distance (+64%). This finding is consistent with the significantly increased proportion of time spent in the dark compartment compared to controls (Figure 10C). A lower number of transitions between the two compartments showed impairments in exploratory behavior of both ABCB1 and ABCC1 transporter-deficient animals. The number of transitions was significantly decreased by 59% (ABCB1^0/0^) and 49% (ABCC1^0/0^) compared to controls (Figure 10D). Rearing activity was differentially altered within the different maze compartments. While ABCC1-deficient mice showed significantly decreased rearing activity in the open-field compartment (−67%; Figure 10E) and no difference in the dark compartment (Figure 10F), mice lacking ABCB1 did not show alterations in rearing activity in the open field (Figure 10E), but increased activity in the dark compartment (+71%; Figure 10F).

## Discussion

ABC transporter function has been proposed to influence hematopoietic and neuronal stem cell proliferation and differentiation *in vitro* [31]. Here, we demonstrate for the first time that the loss of ABC transporter function also leads to specific impairments in neurogenesis and neurofunctional changes *in vivo*. We show that the deficiency of the ABC transporter ABCB1 significantly affects stem cell homeostasis *in vivo*, while these effects do not appear to be based upon cell-intrinsic effects due to unaffected NSPC differentiation *in vitro*. Furthermore, we were able to determine that the lack of ABCC1, a transporter not yet recognized in the setting of NSPC functions, leads to a significant decline in the neurogenic differentiation of NSPCs during disease states such as traumatic brain injury. With regard to cognitive functions, we found that ABC transporter status influences the levels of overall activity and anxiety.

Recent *in vitro* studies revealed ABCB1 and ABCG2 expression in proliferating neuronal progenitors. The expression disappeared during differentiation, thus suggesting a role of the transporters in maintaining the cells in a specified state [29], [30], [48]. With this in mind, we sought to find quantitative impairments in the neuronal stem and progenitor cell pool. However, ABC transporter-deficiency did not significantly alter the Sox2^+^ NSPC pool. Rather, the absence of ABCB1 impaired the differentiation of NSPCs into the neuronal lineage, as detected by significantly decreased amounts of DCX^+^ and calretinin^+^ cells. Although ABCG2 was found to be expressed in NSPCs [30], in our analysis, its deficiency only slightly altered neurogenic functions in ABCG2-deficient mice *in vivo*. Analyzing the BrdU^+^ fraction of Sox2^+^ cells, we found impaired proliferation in the ABC transporter-deficient mice. For ABCG2^0/0^ and ABCC1^0/0^ mice, the BrdU^+^ fraction of calretinin^+^ cells increased significantly. Therefore, it is likely that NSPCs lacking one of the analyzed ABC transporters experience a higher turnover because they are not able to stabilize their stem cell character, thus supporting the view that ABC transporters are involved in NSPC homeostasis.

The impairment of neurogenic differentiation in ABCC1 transporter-deficient mice was disclosed using an induction model in which the brain was stressed by acute traumatic injury. Thus, disease states demanding increased regeneration potential help to uncover functions of ABC transporters beyond their known roles in normal cell physiology. Indeed, such disease-related functions are of broad interest in that they may be linked to neurodegenerative diseases, stroke or the effects of brain aging. With regard to aging, it is important to consider the physiological function of ABC transporters not only at barriers and epithelia, but also in the brain's NSPC pool. The ABC transporters convey a wide spectrum of biological and metabolic substrates [2], [3], [4].

The impairment of ABC-mediated excretion across the BBB might alter the microenvironment of the stem cell niche, which is located directly between the vascular and the ventricular system. The interaction of the NSPCs with both systems thereby implies changes in the NSPC's homeostasis due to altered ABC transport [49]. In the absence of sufficient ABC transport, metabolites and chemical compounds can reach functional and/or pathogenic concentrations within the cells, and thus influence the NSPC pool or its differentiation behavior. For instance, it has been shown that endothelial cells are able to generate soluble factors that can influence the differentiation of NSPCs *in vitro* [50]. Assuming that some of these factors are substrates for ABC transporters, their diminished elimination by transporters could adversely influence both the microenvironment of the stem cell niche and cellular homeostasis, thereby altering NSPC functions.

Our investigations of the proliferation capacity and ability to differentiate into neuronal lineage *in vitro* did not support the idea of a cell-intrinsic function of any of these ABC transporters, directly influencing the cell fate [29], [30], [31]. While we found that NSPCs appear to benefit from the absence of ABCG2 expression, as evidenced by higher cell proliferation, our differentiation experiments did not reveal any impairment due to ABC transporter-deficiency. In particular, the increased proliferation capacity of NSPCs lacking ABCG2 expression does not easily integrate into the observations made by analyzing neurogenic functions in the DG. Rather, this experiment supports the idea of ABC transporter-deficiency induced changes in the global microenvironment of the brain. Furthermore, this altered microenvironment could lead to the observed functional changes in neurogenesis and even behavior.

While our mRNA expression analyses confirmed that all ABC transporters investigated are expressed in NSPCs (except the murine ABCB1 subtype “a”), we also found evidence of possible co-regulation of the expression levels of specific ABC transporters. While the lack of ABCB1b expression augmented both ABCG2 and ABCC1 expression, in contrast, the loss of ABCG2 only had a small lowering effect on ABCB1b expression, and deficiency of ABCC1 did not affect expression of ABCB1b or ABCG2. While previous studies have not found regulatory mechanisms between ABC transporter expression by analyzing whole brain homogenates [51], [52], we show NSPC-specific regulatory mechanisms in ABC transporter mRNA expression for the first time. It is conceivable that these regulatory effects account for the altered proliferation capacity in ABCG2^0/0^ strains due to altered ABC transporter expression profiles, increasing the cells' capability to cope with the cell culture environment. These cell-intrinsic effects are so specific to environmental parameters of the *in vitro* situation that this effect may be simply overwhelmed by the far-ranging effects of altered brain homeostasis due to ABC transporter-deficiency, leading to the effects observed *in vivo*.

The CCI model impairs the relatively isolated (privileged) state of the brain by disrupting the blood-brain barrier. Following trauma, metabolites from the bloodstream can directly access and influence the previously protected stem cell pools. The excretion functions of ABC transporters are also highly taxed to maintain cellular homeostasis. In our experiments, the effects of ABCC1-deficiency became most evident after CCI. Interestingly, ABCC1 has been discovered recently to play an important role in the excretion of toxic Aβ peptides in Alzheimer's disease models via the brain's barriers [13]. In addition to its accumulation in amyloid plaques, Aβ monomers and oligomers have been recognized to directly impair NSPC function [53]. Since the present study shows that ABCC1 function also is important for stem cell differentiation during disease states, it appears that ABC transporters perform dual functions: i) clearing the brain of toxic metabolites, and ii) preserving the microenvironment of the stem cells in their niche. Both functions are necessary to protect against age-related neurodegenerative processes.

Inasmuch as the lack of ABC transporters affects cellular function in the brain, we hypothesized that behavioral integrity might be compromised as well. Therefore, we assessed the effects of ABC transporter deficiency on specific behaviors by using the Y-maze, elevated plus-maze and light/dark box to assess overall activity, exploratory drive and anxiety. We detected marked differences in both activity and exploratory behavior in ABCB1- and ABCC1-deficient mice. The decreased activity might partially reflect the increased anxiety levels that were detected in the elevated plus maze. Notably, light/dark box tests revealed complex behavioral alterations in those mice. While both ABCB1^0/0^ and ABCC1^0/0^ mice show stronger avoidance of the light area (stress) than did controls, ABCB1^0/0^ mice displayed reduced fear/enhanced exploratory behavior under non-stress conditions (dark area) and normal behavior in the stressful environment. Therefore, ABCC1^0/0^ mice kept a normal phenotype under non-stress conditions, but intensified anxiety in the light area (stress). These observations indicate the need for further studies that will elucidate the complex behavioral changes in these strains.

Deficiency in ABC transporter expression somehow seems to minor effect brain ontogenesis in layer I and layer VI (layers with least cellular density and mostly interconnecting axons and dendrites) according to the altered cortical architecture in ABCB1 and ABCC1 transporter-deficient mice. Whether these changes are primary or secondary due to changes in the cytoarchitecture of other layers remains unclear since the total number of neurons is not changed (Figure 7B). In addition to ontogenetic changes in brain structure, it seems likely that ABC transporters alter the biological milieu within the cells/brain. Steroids, for example, are substrates for ABC transporters [54] that are able to alter anxiogenic functions in specific sets of hippocampal neurons [55]. Neurogenesis may contribute to certain behaviors, such as enabling spatial learning in orientation tasks [56]. Because the analyzed ABC transporter-deficient mice have an FVB/N background with a known retinal degeneration leading to visual deficits within the first 30 days of life [57], [58], we were not able to analyze the animals' abilities in spatial orientation tasks based on visual cues. Regarding performance on the other tasks, the visual impairment should not be of consequence, due to the fact that close-range orientation is based also on tactile stimuli [59]. The mice are able to orient within the narrow corridors of the Y-maze and easily recognize the open field and abyss surrounding the open arms of the plus-maze, as shown in previous studies [60].

In summary, the diverse ABC transporter subfamily members play important roles in physiologic and disease states of the brain. Alteration of their function due to genetic predisposition (e.g. promoter activity), slight structural variations (e.g. amino acid exchanges), or transport activity (e.g. by drugs or metabolites) may lead to early or prolonged effects on stem cell homing and differentiation. ABC transporters thus help to regulate two functions that are important for maintaining the integrity of the brain during aging and neurodegeneration – they export specific agents via the brain's barriers, and they also maintain the integrity and functionality of the stem cell pool. The large number of ABC transporter-modulating drugs that are used for the treatment of chronic diseases suggests the feasibility of testing such agents as potential modulators of neurodegeneration in acute and chronic disorders.

## Materials and Methods

### Animals

FVB.129P2-Abcg2^tm1Ahs^ N7 (ABCG2^0/0^), FVB.129P2-Abcb1a^tm1Bor^Abcb1b^tm1Bor^ N12 (ABCB1^0/0^), and FVB.129P2-Abcc1a^tm1Bor^ N12 (ABCC1^0/0^) mice were purchased from Taconic Farms (Denmark) [28], [61], [62]. All mice were housed under a 12 h/12 h light/dark cycle at 22°C with free access to food (SNIFF, Germany) and water. All protocols involving the use of animals were approved by the local authorities (LALLF M-V TSD 7221.3-1.1-035/10 and LALLF M-V TSD 7221.3-1.1-024/11).

### CCI-Surgery and BrdU injections

For each group (FVB controls, ABCB1^0/0^, ABCG2^0/0^, ABCC1^0/0^), 6 mice (3 males, 3 females) were used for controlled cortical impact (CCI)-surgery at the age of 90 days. Animals were anesthetized using a ketamine/xylazine mixture (1.9 mg/ml ketamine; 0.5 mg/ml xylazine) at 20 µl per gram bodyweight. Animals were placed in a stereotactic frame and a midline incision was made to expose the skull. The skull was opened using a drill (ø 2 mm) at the position bregma −2.0 mm anterior and bregma −2.0 mm lateral. The impact device used for the CCI consisted of a 1.2 mm diameter steel cannula with a deformed tip. The impact device was driven 2.2 mm into the brain above the hippocampus. After surgery, the wound was closed using suture clips [38], [39]. To analyze the proliferation capacities, a daily dose of 50 µg BrdU per gram body weight was administered intraperitoneally for 7 consecutive days prior sacrifice.

### Immunofluorescence and immunohistochemistry

#### Immunofluorescence

All animals were killed by cervical dislocation and immediately transcardially perfused with 20 ml of PBS followed by 20 ml of buffered, 4% paraformaldehyde (PFA) as described previously [63]. The brains were extracted and post-fixed in 4% PFA overnight and consecutively immersed in 15% sucrose and 25% sucrose solutions, each step overnight. Whole brains were mounted and frozen in cryo media (OCT Compound, Tissue Tek). We chose a specific range of the hippocampus (bregma −1.5 mm to bregma −2.2 mm) and cut (Leica CM3050 S) serial coronal sections (16 µm) within this 720 µm range from whole brains and separated every third section alternately to one of three containers for the different immunofluorescence staining protocols. The entire number of sections was 45, which yielded 15 sections per group. For each protocol, 6 slices were taken randomly from the respective pool and were stained immunofluorescently. Cells were counted if within the subgranular zone or no more than two cell diameters away [64] in both hemispheres. The spacing of at least two sections between the evaluated sections of each marker group maintains the key consideration of unbiased stereological cell quantification that no cell will occur in more than one section and therefore would not be counted twice.

To reveal BrdU labeling, the brain sections were pre-incubated in 1.5 M HCl for 30 min at 37°C to denature the DNA. Then, the slices were washed three times in PBS before blocking for an hour in 5% chicken serum in PBS with 0.5% Triton X-100. The sections were incubated free-floating with the primary antibodies anti-BrdU (1∶600; Sigma clone BU-33), anti-Sox2 (1∶400; Santa Cruz clone Y-17), anti-DCX (1∶400; Santa Cruz clone C-18) and anti-calretinin (1∶2000; Santa Cruz clone N-18) overnight in blocking buffer at 4°C in the appropriate combinations of BrdU/Sox2, BrdU/DCX and BrdU/calretinin. Incubation with fluorescence-labeled secondary antibodies (1∶2000 anti-mouse Cy2; 1∶2000 anti-goat Cy3; Dianova, Germany) was performed for 2 hours at room temperature after washing in PBS. The slices were covered with DePex (Serva Electrophoresis, Germany) and visualized using a Zeiss LSM 700 microscope. Cells were counted if they were within the subgranular zone (SGZ) and excluded if they were more than two cell diameters from the SGZ [64]. For quantification of different cell populations, the reviewers remained incognizant of the experimental groups. The labeled cells in the dentate gyrus of each hemisphere were counted in 3 to 6 slices per animal and marker combination. Six animals per group (3 males, 3 females) were analyzed.

#### Immunohistochemistry

Brains were harvested as described above and post-fixed for at least 24 hours. The tissue was paraffin-embedded, and 4 µm-thick coronal sections were cut. Neurons were stained using the NeuN primary antibody (1∶1000; Millipore clone MAB377) on a Bond-Max™ (Leica, Germany) automated staining system and the Bond™ Polymer Refine Detection kit (Leica, Germany).

### Digitization and analyses of NeuN-stained brain slices

Brain slices were digitized using a MIRAX Midi slide scanner (Zeiss Microsystems, Germany). The scanned brain slices were processed using a semi-automatic software macro programmed within the AxioVision software package (Zeiss Microsystems, Germany) to separate the stained neurons from the rest of the cortical tissue. Specific regions of interest (ROI) were defined in the cortex and used for further digital processing and area normalization [41]. Furthermore, the NeuN stained sections were used to determine the size of the cortical layers with the AxioVision software package (Zeiss Microsystems, Germany). Therefore, we measured the thickness of layer I, layer II-IV (combined), layer V and layer VI according to Manuel *et al.* [42]. On each brain section two measurements were performed approximately in the lateral parietal association cortex and the barrel field of the primary somatosensory cortex. The average of both measurements built the value for the according slice. The average value for a distinct animal was calculated of the values of its two associated hemispheres. For the analyses of cortical cytoarchitecture 5–7 animals were analyzed for each strain.

### Primary NSPC culture

Brains were extracted under sterile conditions from male animals aged between 50 and 80 days. Harvested brains from 4 to 6 animals were transferred into ice-cold aCSF-media [65] to cut 2 mm thick coronal slices 6–8 mm behind the olfactory bulbs of each brain. The tissue around the lateral ventricle was harvested from the slices and was enzymatically digested for 10 min at 37°C using a Trypsin/EDTA solution (GIBCO, Invitrogen Inc.). After additional mechanical dissociation, cells were seeded in neurosphere culture media (NeuroCult®, STEMCELL Technologies Inc.) supplemented with EGF (20 ng/mL) and FGF (10 ng/mL) (Peprotech Inc., USA) for 7 days until passaging after supplier's instructions. Cell lines were passaged at least four times before use in proliferation or differentiation assays [66].

#### Proliferation assay

For measuring proliferation capacity, cells (5×10^4^ cells/cm^2^) were seeded for 24 h on Matrigel-coated coverslips in 24-well plates under proliferation conditions. BrdU (10 µM) was administered 1 h prior to fixation with paraformaldehyde (4% in PBS). Labeling of incorporated BrdU and cell nuclei using DAPI was performed according to the description in the immunofluorescence section. Stained coverslips were mounted in DePex (Serva Electrophoresis, Germany) on glass slides and cells were visualized using a Zeiss laser scanning microscope LSM 700. The relative proportion of BrdU^+^ cell nuclei against the overall number of DAPI^+^ nuclei was used to compare the proliferation capacity. The number of independent experiments was n≥11 for each strain.

#### Differentiation assay

Cells were seeded on Matrigel-coated coverslips (1×10^5^ cells/cm^2^) and were allowed to differentiate under withdrawal of growth factors in differentiation media (NeuroCult® Differentiation kit, STEMCELL Technologies Inc.). After 3 days, cells were fixed for 10 minutes with paraformaldehyde. The coverslips were incubated with a primary antibody against Tuj1 (1∶500, Covance) in blocking buffer at 4°C. Incubation with fluorescence-labeled secondary antibody (1∶2000 anti-mouse Cy3; Dianova, Germany) was performed for 2 hours at room temperature after washing in PBS. The coverslips were mounted in DePex on glass slides and were visualized using a Zeiss LSM 700 microscope. The proportion of Tuj1^+^ cells against the whole number of DAPI^+^ nuclei was used to compare the proportion of newly differentiated neurons [53]. The number of independent experiments was n≥12 for each strain.

#### ABC transporter expression analysis using TaqMan® assays

Briefly, RNA was extracted from proliferating neurosphere cultures at day three after passage using the Trizol method. RNA was stored for later analysis at −80C° in elution buffer. 10 pg RNA were directly used for combined reverse transcription (One-Step RT-PCR, Applied Biosystems) and realtime quantitative PCR (TaqMan® assays, Applied Biosystems) in a single tube using a Rotorgene 6000 (Qiagen) according to the supplier's instructions. Multiplex reactions were performed by simultaneously quantifying the FAM-labeled internal control (GAPDH) and the VIC-labeled test sequence (ABC transporters). Relative expression levels were calculated using the ΔΔC_T_ method [67]. The following TaqMan® assays were purchased from Applied Biosystems: ABCB1a (cat# Mm00440761_m1), ABCB1b (cat# Mm00440736_m1). ABCG2 (cat# Mm00496364_m1), ABCC1 (cat# Mm00456156_m1) and GAPDH (cat# Mm99999915_g1). The number of analyzed NSPC RNA samples is n = 3 for each group.

### Statistics

For quantification of different cell populations in stained brain slices or cell culture, the reviewers remained incognizant of the experimental groups. For all quantifications, statistical significance (p≤0.05) was determined using unpaired t-tests with Welch's correction (Graph Pad Prism 5).

### Behavioral tests

#### Y-maze

Mice were tested in random order in a symmetrical Y-shaped maze (TSE Systems). The dimensions of each arm were 325 mm (length), 85 mm (width) and 150 mm (height). Mice were put into the middle of the maze and were allowed to move freely throughout the maze. Their movement path was recorded with a camera during the test duration of 5 min and analyzed with the *VideoMot* software package (TSE Systems). The following parameters were assessed: running distance, number of arm entries, and the percentage of alternation [68]. At least 10 animals per group were investigated.

#### Elevated Plus-Maze

Mice were tested randomly in an elevated Plus-maze (TSE Systems). The dimensions of the arms were: length 300 mm and width 50 mm; the maze was elevated 50 cm above the ground. Two opposing arms were either open or enclosed by a wall of 150 mm height. To start, the mice were placed in the center of the maze and allowed to move freely throughout the entire maze. The path was recorded with a camera during the test duration of 5 min and analyzed with the *VideoMot* software package (TSE Systems). The following parameters were assessed: number of closed arm entries, open arm entries, the mouse's speed in each compartment, the time spent in each of the arms, and the time spent in the central area [69]. At least 9 animals per group were investigated.

#### Light/Dark box test

Mice were tested randomly in a Light**/**Dark-Box (TSE Systems). The maze setup consisted of a box (500 mm×500 mm×400 mm) that is divided into a darkened (1/3^rd^) and an illuminated compartment (2/3^rd^, illuminated at ∼400 Lux) connected by a door (60 mm×60 mm). The mice were set in the middle of the illuminated compartment facing away from the door and were allowed to move freely within the two compartments of the maze for 5 min. The course of the animal's movement was recorded using an infrared camera and infrared light transilluminating the whole maze from below. The software package *VideoMot* (TSE Systems) was used to analyze parameters such as duration of stay per compartment, distance travelled per compartment, transitions between compartments, and number of rearing in each compartment [70]. At least 9 animals per group were investigated.
